# Label-Free Optical Technologies for Middle-Ear Diseases

**DOI:** 10.3390/bioengineering11020104

**Published:** 2024-01-23

**Authors:** Zeyi Zhou, Rishikesh Pandey, Tulio A. Valdez

**Affiliations:** 1 School of Medicine, Stanford University, Palo Alto, CA 94305, USA; 2 Department of Biomedical Engineering, University of Connecticut, Storrs, CT 06269, USA; 3 Department of Otolaryngology, Stanford University, Palo Alto, CA 94304, USA

**Keywords:** label-free imaging, middle-ear disease, optical technology

## Abstract

Medical applications of optical technology have increased tremendously in recent decades. Label-free techniques have the unique advantage of investigating biological samples in vivo without introducing exogenous agents. This is especially beneficial for a rapid clinical translation as it reduces the need for toxicity studies and regulatory approval for exogenous labels. Emerging applications have utilized label-free optical technology for screening, diagnosis, and surgical guidance. Advancements in detection technology and rapid improvements in artificial intelligence have expedited the clinical implementation of some optical technologies. Among numerous biomedical application areas, middle-ear disease is a unique space where label-free technology has great potential. The middle ear has a unique anatomical location that can be accessed through a dark channel, the external auditory canal; it can be sampled through a tympanic membrane of approximately 100 microns in thickness. The tympanic membrane is the only membrane in the body that is surrounded by air on both sides, under normal conditions. Despite these favorable characteristics, current examination modalities for middle-ear space utilize century-old technology such as white-light otoscopy. This paper reviews existing label-free imaging technologies and their current progress in visualizing middle-ear diseases. We discuss potential opportunities, barriers, and practical considerations when transitioning label-free technology to clinical applications.

## 1. Introduction

Optical technology has received considerable attention in clinical applications in recent decades. Prominent examples include narrow-band colonoscopy in colorectal neoplasia and polyp diagnosis, blue-light cystoscopy for urological procedures, optical coherence tomography in ophthalmology clinics, and fluorescence-guided surgery in the operating room [1,2,3,4,5].

Although the use of fluorescence-based contrast agents has shown enormous promise in biomedical applications, their clinical application is impeded by toxicity concerns, thereby posing a hurdle for regulatory approval [6]. Consequently, techniques that exploit the intrinsic molecular contrast or morphology of biological specimens offer an interesting alternative to contrast-based approaches. This is especially critical for translation to clinical applications, where it is simply not feasible to introduce toxic fluorescent markers and probes to patients. In middle-ear imaging, this is even more important as ototoxicity is always a major concern. The sensitivity to tissue microstructures, variable penetration depths, and rapid imaging speed and analysis powered by artificial intelligence (AI) suggest that label-free optical-based imaging would be of great utility in the middle-ear domain.

The label-free optical techniques may be broadly classified into two groups; namely, spectroscopic and interferometric approaches. The spectroscopic techniques rely on the optical properties of biospecimens and changes in the optical properties due to disease progression are utilized to detect and diagnose the disease. Different kinds of optical properties such as absorption, emission, and scattering are measured using different techniques such as diffuse reflectance spectroscopy, infrared spectroscopy, autofluorescence spectroscopy, and Raman spectroscopy. These techniques have been investigated for different applications in middle-ear disease and, of course, have their individual strengths and shortcomings; their application depends on the target application. In some cases, two complementary techniques have been combined to increase the spectral information content. Interferometric techniques such as optical coherence tomography are based on the scattering from samples that are measured with the help of an interference pattern. Spectroscopic techniques encode molecular-specific information; interferometric-based approaches measure the bulk properties of tissue.

In this review paper, we aim to summarize the current advancements in label-free imaging technologies for middle-ear diseases for both engineers and clinicians. We first briefly outline the principles of existing label-free optical technologies, including autofluorescence imaging, diffuse reflectance imaging, Raman spectroscopy, SWIR imaging, and optical coherence tomography. Then, we review their application in various middle-ear diseases in both ex vivo and in vivo settings (Table 1). We then proceed to discuss the potential opportunities for label-free technology, which includes bacterial identification and integration with machine learning and artificial intelligence algorithms. Lastly, we describe the various barriers and considerations necessary for clinical translation in middle-ear diseases.

## 2. Label-Free Optical Techniques

### 2.1. Autofluorescence Imaging

Autofluorescence (AF) spectroscopy measures emissions from the natural fluorophores in biospecimens. These fluorophores include collagen, elastin, nicotinamide adenine dinucleotide (NADH), flavins, carotenoids, and porphyrins. Compared with exogenous contrast-based fluorescence spectroscopy, AF signals are feeble and less specific. However, the availability of intense LEDs in the near-infrared (NIR) to ultraviolet-C (UVC) region, advancements in sensor technology, and the advent of new AI approaches have enabled the optimal and selective excitation of these chromophores to utilize the emission properties for biomedical applications. AF imaging marries the fluorescence information with the microtextural information (morphology) of biospecimens and provides a higher information content. AF imaging is routinely used in ophthalmic clinics for the diagnosis and monitoring of retinal diseases and its application for the surgical detection of the parathyroid gland and a variety of cancers is currently being realized [31,32,33,34].

### 2.2. Diffuse Reflectance Imaging

Diffuse reflectance spectroscopy (DRS) measures both the differential absorption and scattering properties of the specimens. The different molecular absorbers include amino acids, hemoglobin, melanin, collagen, carotenoids, and porphyrins. Owing to the recent advancements in hyperspectral camera technology, multispectral images provide sufficient differential information content that can be used for diagnostic purposes or better imaging enhancement. Narrow-band imaging is one of the modalities that has gained clinical adoption in various clinical fields such as gastroenterology and otolaryngology. DRS not only measures the concentrations of constituent absorbers but also provides information from tissue scatterers such as nuclei and glands.

### 2.3. Raman Spectroscopy

Unlike AF and reflectance spectroscopies, Raman spectroscopy (RS) measures the inelastic scattering of the molecules. The Raman spectrum, which provides a plot intensity of inelastically scattered light vs. the frequency (wavenumber) of light, provides molecular fingerprinting information and forms the basis for tissue discrimination. As the Raman signal is proportional to the concentration, Raman spectroscopy can be used to monitor relevant biomolecules/biomarkers in a biospecimen. Technological advancements over recent decades such as the availability of compact NIR lasers, the development of fiber-optic Raman probes, high quantum efficiency charge-coupled devices (CCDs) in the NIR range, and high-throughput (yet miniaturized) spectrometers have enabled RS to be useful for clinical use. Further, Raman imaging, which combines the molecular-specific information of RS and spatial attributes of light microscopy, has enabled the acquisition of high-fidelity Raman images at cellular and subcellular scales.

### 2.4. SWIR Imaging

The scattering and absorption of tissue limit the imaging/sensing of deep layers of tissue. The use of shortwave infrared light (SWIR), which typically extends from 1000 to 2000 nm, enables the imaging of deep layers of tissue and the ability to non-invasively interrogate subsurface tissue features. Reduced scattering enables us to appreciate changes in the absorption characteristics of tissue constituents such as water, collagen, and lipids. Consequently, enhanced sensitivity to these biomolecules in SWIR enables a better characterization of concentration changes with a higher penetration depth. However, the cost-prohibitive nature of non-silicon semiconductor detector arrays and limited/restricted access have slowed its application in biomedical research. Fortunately, these limitations are less applicable now, presenting the opportunity to use SWIR. This presents SWIR as an ideal technique to see inside the middle-ear space through the intact tympanic membrane. This modality can be used as label-free reflectance or combined with molecular probes and dyes.

### 2.5. Optical Coherence Tomography

Optical coherence tomography (OCT) uses low-coherence light to capture images by utilizing the interference properties of biological tissues at a micrometer resolution. This uses a long-wavelength NIR light, allowing a higher penetration depth into the tissue, like SWIR. Unlike SWIR and other spectroscopic techniques, OCT provides bulk scattering information from the tissue and lacks molecular-specific features. OCT is now routinely used in ophthalmology, with active research on applications in other fields such as arterial imaging and guided biopsies for multiple cancers [35,36,37,38,39]. More recently, OCT has been proposed as a modality to help in the diagnosis of otitis media [25].

## 3. Applications in Disease Diagnosis and Visualization

### 3.1. Ex Vivo Application

#### 3.1.1. Tissue Diagnosis

White-light otoscopy remains the gold standard for the diagnosis and detection of many middle-ear pathologies; however, its interpretation is subject to significant intraobserver variability [40]. Tissue diagnosis in an ex vivo setting is one of the applications for which label-free technology has been implemented for middle-ear diseases, providing not only morphological information but also biochemical information about the samples.

**Figure 1:**
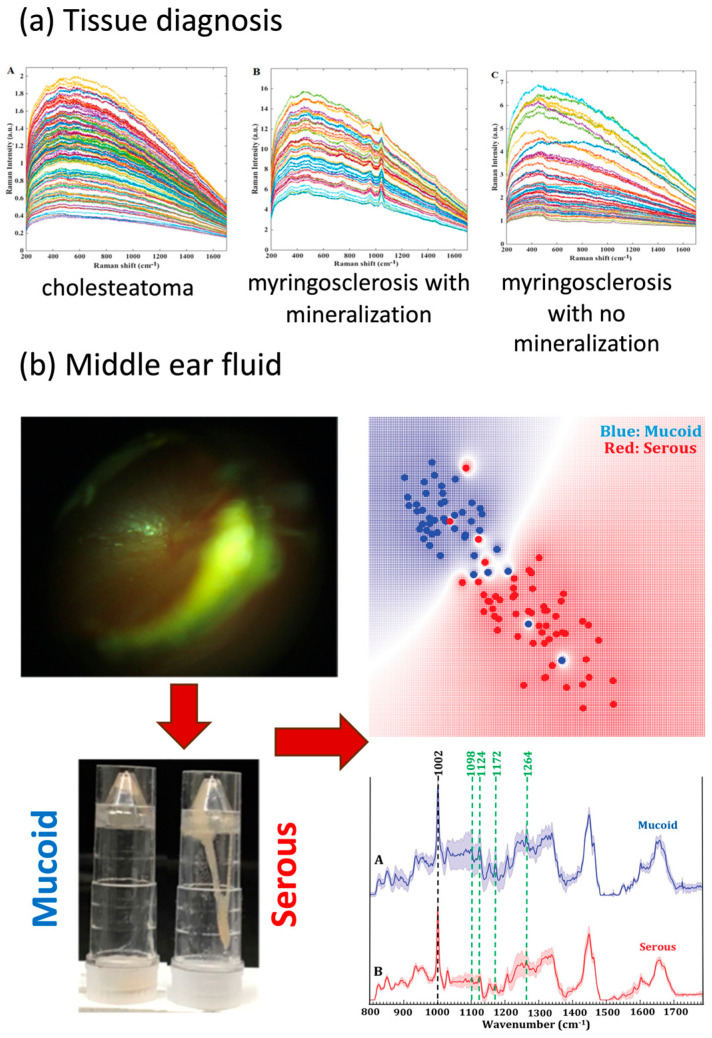
Ex vivo applications of label-free technologies in middle-ear space. (**a**) Differences in Raman spectra between cholesteatoma, myringosclerosis with mineralization, and myringosclerosis with no mineralization. Reprinted from [8] *Discerning the differential molecular pathology of proliferative middle ear lesions using Raman spectroscopy* by R. Pandey, 2015, Scientific Reports, 5(1): 13,305 (DOI: 10.1038/srep13305). CC BY 4.0. (**b**) Differentiating middle-ear fluids using Raman spectroscopy. Adapted from [13] *Differential diagnosis of otitis media with effusion using label-free Raman spectroscopy: A pilot study* by R. Pandey, 2018, J Biophotonics, 11(6): e201700259 (DOI:10.1002/jbio.201700259). Copyright 2018 by John Wiley and Sons, NJ, USA.

Raman spectroscopy has been reported to be able to distinguish between the molecular pathology of different middle-ear lesions. Specifically, applications of Raman spectroscopy have been shown to be able to provide a label-free, real-time, and molecular-specific probe for the diagnosis and differentiation of cholesteatoma and myringosclerosis in ex vivo middle-ear lesion samples [8]. The molecular specificity of Raman spectroscopy has allowed researchers to distinguish between cholesteatoma, myringosclerosis with mineralization, and myringosclerosis without mineralization by identifying robust spectral markers of molecules unique to each lesion such as keratin, collagen, calcium, and silica (Figure 1a). The sensitivity and specificity of this classification technique in that study were 95.48% and 99.06%, respectively. Zou et al. showed that anti-Stokes Raman spectroscopy (CARS) microscopy with a combination of two-photon excited fluorescence (TPEF) microscopy and second-harmonic generation (SHG) microscopy could differentiate between cholesteatoma and chronic otitis media in both middle-ear tissue and tympanic membrane samples based on the different enrichments of lipids, protein, and collagen [7]. Both studies demonstrate the ability of Raman-based imaging techniques to differentiate middle-ear pathologies such as chronic otitis media and cholesteatoma based on their unique biochemical composition in an ex vivo setting, indicating great potential for both diagnosis and intraoperative surgical guidance.

Wisotzky et al. reported the optical properties of cholesteatoma and bone. They demonstrated differences in absorption and the scattering coefficient in the wavelength range of 250 nm to 800 nm within samples collected from surgeries. This work demonstrated great potential for the development of an intraoperative image guidance system based on multispectral imaging to differentiate cholesteatoma from surrounding middle-ear mucosa and bone tissue based on their unique optical properties; this was then developed by the same group and is discussed below [9].

Yang et al. developed and prototyped an imaging system able to differentiate cholesteatoma tissue from middle-ear tissue excised from patients using 405 and 450 nm illumination. Cholesteatoma tissues had discernible autofluorescence whereas middle-ear mucosa did not, taking advantage of the autofluorescence properties of keratin and flavin adenine dinucleotide [10]. This imaging system combined a traditional endoscope with a 495 nm longpass filter and an RGB camera, which particularly illustrated the great potential for the application of such a label-free imaging system in intraoperative guidance for cholesteatoma removal.

Enzian et al. reported using fluorescence lifetime imaging microscopy (FLIM) on cryopreserved human middle-ear tissue samples and they were able to observe different fluorescence lifetimes in the 500–575 nm emission range with 473 nm excitation. This represents a promising and different technique, taking advantage of the autofluorescence properties of molecules such as collagen, elastin, and keratin present in the middle ear [11].

#### 3.1.2. Middle-Ear Fluid

Carr et al. looked at the detection of phantom middle-ear fluid in a 3D-printed middle-ear model using a shortwave infrared (SWIR)-enhanced otoscope, which showed a significantly better light absorption beyond 1300 nm of middle-ear fluid compared with traditional white-light illumination. This allowed a more accurate detection of the presence of middle-ear fluid, enabling treatment to be guided accordingly. In this manuscript, the authors discussed the potential to discern between mucoid and serous fluid by measuring the attenuation at different wavelengths but did not conduct experiments [12].

Furthermore, a Raman Spectroscopy approach with multivariate analysis of spectral patterns was shown, in a proof-of-concept study, to distinguish components in middle-ear effusion based on characteristic markers [13]. Specifically, in this study, Pandey et al. demonstrated the ability to discern between serous or mucoid fluid. This has great clinical significance for otitis media with effusion as serous fluid usually represents a self-limiting potential, while mucoid-based fluid often requires surgical intervention (Figure 1b). This provides great potential for the development of more accurate non-invasive diagnostic tools of middle-ear effusion that could guide more precise treatment plans.

#### 3.1.3. Tympanic Membrane Visualization

Optical coherence tomography (OCT) has been shown to be able to non-invasively visualize the tympanic membrane and middle-ear structures across the tympanic membrane without perforating the tympanic membrane in harvested cadaver middle-ear tissues. Pitrius et al. showed that OCT could image the tympanic membrane, middle ossicles, nerves, and stapedial tendons in a cross-sectional fashion with high resolution in real time [14]. Nguyen et al. reported a portable imaging system of low-coherence interferometry (LCI) combined with a standard otoscope that was able to both detect and measure middle-ear biofilms in an animal model. They also reported a novel classification algorithm that was able to detect biofilms with a sensitivity of 87% and specificity of 90% [15]. This technology shows great promise for the diagnosis of chronic otitis media and monitoring the antibiotic response.

SWIR imaging has been shown to have deeper tissue penetration and less light scattering compared with traditional imaging methods in the visible or near-infrared windows. SWIR allows a better image contrast and visibility of deeper middle-ear structures such as the ossicular chain and round window niche through the thin tympanic membrane [12]. Better visualization across the tympanic membrane and middle-ear ossicles could have great potential for more accurate diagnosis, treatment selection, and surgical guidance for procedures such as ossicular reconstructions and the monitoring of electrode insertions in cochlear implantations.

### 3.2. In Vivo Application

In addition to the great potential label-free imaging has shown in studying middle-ear disease in an ex vivo setting, many studies have been conducted using humans by applying label-free imaging in an in vivo setting. Some devices have already obtained FDA clearance, with the ultimate goal of clinical adoption and application.

#### 3.2.1. Tissue Differentiation and Visualization of Tympanic Membrane

**Figure 2:**
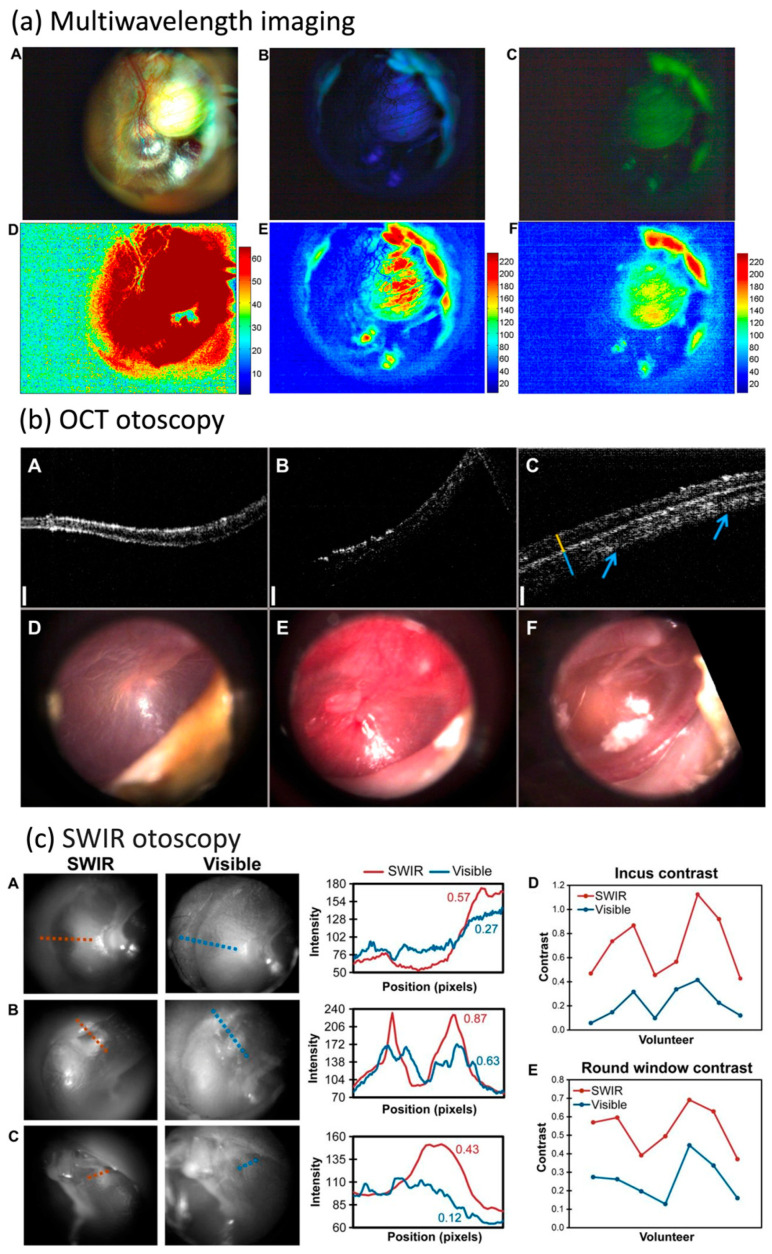
In vivo applications of label-free technologies in middle-ear space. (**a**) White-light vs. multiwavelength imaging of cholesteatoma on tympanic membrane. A–C show raw images: white light image (A), fluorescence image at 405 nm excitation (B) and at 450 excitation (C), D–F show corresponding denoised images. Reprinted from [17] *Multiwavelength Fluorescence Otoscope for Video-Rate Chemical Imaging of Middle Ear Pathology* by T. Valdez, 2014, Analytical Chemistry, 86, 10454−10460 (DOI: 10.1021/ac5030232) Copyright 2014 by American Chemistry Society. (**b**) Measurement of tympanic membrane via OCT in A–C, A for normal tympanic membrane, B for acute otitis media, C for chronic otitis media. D–F are corresponding otoscope image. Reprinted from [25] *Noninvasive Depth-Resolved Optical Measurements of the Tympanic Membrane and Middle Ear for Differentiating Otitis Media* by G. Monroy, 2015, Laryngoscope, 125(8): E276–E282 (DOI:10.1002/lary.25141) Copyright 2015 by John Wiley and Sons. (**c**) White-light vs. SWIR imaging of cholesteatoma on tympanic membrane. A demonstrates the round window, B and C demonstrates the incus of two different volunteers respectively. D and E shows the comparison of incus (D) and round window (E) across all volunteers. Reprinted from [12] *Using the shortwave infrared to image middle ear pathologies* by J. Carr, 2016, Proc Natl Acad Sci, 113(36): 9989–94 Copyright 2016 by National Academy of Sciences.

**Figure 3:**
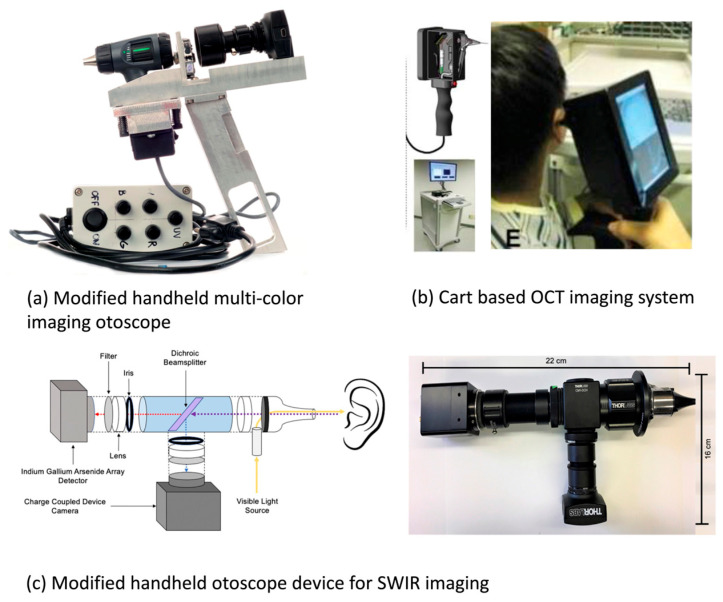
Label-free imaging system and device setup for middle-ear space. (**a**) Modified handheld otoscope device for multicolor reflectance imaging. Reprinted from [16] *Multi-color reflectance imaging of middle ear pathology* in vivo by T. Valdez, 2015, Anal Bioanal Chem., 407(12): 3277–3283 (DOI:10.1007/s00216-015-8580-y). Copyright 2015 by Springer Nature. (**b**) Cart-based OCT system. Adapted from [28] *Optical coherence tomography for advanced screening in the primary care office* by R. Shelton, 2014, J Biophotonics, 7(7): 525–533 (DOI: 10.1002/jbio.201200243). Copyright 2014 by John Wiley and Sons. (**c**) Modified handheld otoscope device for SWIR imaging. Reprinted from [24] *Shortwave infrared otoscopy for diagnosis of middle ear effusions: a machine-learning-based approach* by R Kashani, 2021, Sci Rep, 11(1): 12,509 (DOI: 10.1038/s41598-021-91736-9) CC BY 4.0.

Multicolor imaging is one such promising technique for in vivo applications, with great potential for integration with a traditional otoscopy. Valdez et al. demonstrated a modified otoscope with a custom-designed multiwavelength imaging system that took advantage of the autofluorescence of chemical components such as collagen and keratin in middle-ear pathologies (Figure 2a). The same group later utilized a multicolor reflectance system that offered a better image contrast for tympanic membrane visualization, especially its vascularization. Researchers intraoperatively applied this imaging system to a pilot study of five pediatrics patients with acute otitis media and congenital cholesteatoma (Figure 3a). This approach—with multiple illumination, both blue and green sources—took advantage of the differences in absorption and scattering due to tissue changes in middle-ear diseases. However, more research on the optimization of the best set of illimitation wavelengths for each different pathology and larger clinical studies with larger patient populations are needed for the diagnosis of different pathologies before clinical application [16,17].

Additionally, Tran Van et al. applied multispectral imaging to a cohort of 12 healthy volunteers and found that red-light images, compared with blue-, green-, and white-light images, provided the best contrast to visualize the borders of the tympanic membrane [18]. Wisotzky et al. introduced a multispectral imaging system combined with an endoscope that could switch between narrow-band spectral and broad-band white illuminations. This technique was tested on three surgical patients undergoing cholesteatoma resections. The system was able to correctly identify and visualize the cholesteatoma and produce accurate 3D anatomical reconstructions. This multispectral endoscope system is another label-free imaging system that demonstrates great promise in terms of perioperative planning and intraoperative guidance for cholesteatoma treatments [19]. Multicolor technology has great potential in aiding both the diagnoses and procedural treatments of middle-ear diseases.

SWIR provides another powerful technology with the potential for integration into a traditional otoscopy. Carr et al. first reported a portable SWIR-modified otoscope that was tested on 10 healthy adults. The researchers found that the SWIR otoscope was able to visualize anatomical structures that traditional white-light otoscopes could not detect such as the stapedial tendon, chorda tympani, and round window niche. The SWIR otoscope was also able to provide better contrast to structures visible with a traditional otoscope such as the incus (Figure 2c) [12]. This demonstrates the great potential of SWIR as another label-free imaging technology to be integrated into clinical applications.

Low-coherence interferometry (LCI) and optical coherence tomography (OCT) are also label-free imaging techniques that have shown exciting promise for in vivo use. Djalilian et al. applied an OCT system to a clinical cohort of 10 patients undergoing otologic surgery to intraoperatively image cholesteatoma. The researchers were able to successfully image and differentiate cholesteatoma from both nearby normal tissue and inflamed tissue by identifying the presence of keratin [20]. This demonstrates the great potential for OCT to be implemented in cholesteatoma surgical guidance as the differentiation of cholesteatoma from adjacent tissue using white-light microscopy has traditionally been challenging. Thus, multiple surgeries are often required to eradicate residual or recurrent cholesteatoma [21].

#### 3.2.2. Otitis Media

Otitis media presents another clinical disease area with great potential for label-free optical technology applications, given that the current clinical diagnosis mainly depends on clinical history and white-light otoscopy [41]. Sundberg et al. looked at otitis media with effusion in children, utilizing diffuse reflectance spectroscopy with several erythema detection algorithms. Their group was able to distinguish otitis media with mucous effusion from otitis media with serious effusion by measuring the hemoglobin content in a group of 15 pediatric patients [22].

Valdez et al. reported similar findings when comparing SWIR otoscope with traditional otoscope video recordings in an initial group of 20 pediatric patients during office visits. The SWIR otoscope was also able to better identify middle-ear effusion via better imaging contrast [23]. Kashani et al. reported an improved version of the SWIR otoscope combined with a machine learning algorithm-based approach in a cohort of 30 pediatric patients that was able to intraoperatively and successfully classify middle-ear effusions (Figure 3c) [24].

A series of studies have investigated measuring tympanic membrane thickness in patients using LCI and OCT. Tympanic membrane thickness can provide important clinical information to differentiate healthy tympanic membranes from those with acute and chronic otitis media as the latter often have thicker tympanic membranes due to inflammation and/or biofilm accumulation (Figure 2b) [25]. Nguyen et al. utilized an LCI/OCT setup and examined a cohort of 16 chronic otitis media patients and 4 healthy controls. This approach to identify biofilms showed a sensitivity of 68% and specificity of 98%, with an improvement in sensitivity to 83% in a subcohort of patients with biofilms occupying a significant part of tympanic membrane [26]. A more recent case series study investigated the thickness of the tympanic membrane and associated biofilms in a group of 34 pediatric patients using a modified handheld OCT device. By identifying the thickness of the tympanic membrane and biofilm, researchers were able to differentiate normal, acute, and chronic otitis media [25]. Hubler et al. improved upon the handheld OCT device by combining it with an automated segmentation algorithm, which allowed for the real-time measurement of the thickness of the tympanic membrane in an outpatient setting (Figure 3b) [27,28]. Additionally, Park et al. applied a handheld OCT device to a cohort of 120 patients to evaluate tympanic membrane perforations, tympanic membrane retractions, and postoperative healing of tympanic membranes and middle-ear space, demonstrating the potential clinical benefits of an OCT device complementary to traditional otoscopes from a surgeon perspective [29].

Another emerging direction with great potential is multimodal imaging systems, which incorporate multiple label-free imaging techniques and combine the strengths of various imaging modalities. Cavalcanti et al. reported the development of a smartphone-based multimodal imaging system that offered both autofluorescence and spectral imaging combined with ML analysis. This system was tested on a cohort of 69 patients to differentiate between normal ears, otitis media with effusion, and adhesive otitis media; it achieved an accuracy of 79.63% [30]. The authors suggested that future work focusing on incorporating 3D morphological information would further help to improve the imaging accuracy. The work by Valdez et al. on multiwavelength fluorescence otoscopes and multicolor reflectance imaging could also be combined within the same device to produce a multimodal imaging system to investigate middle-ear pathology in vivo [16,17].

Overall, exciting progress has been made in the testing of in vivo label-free imaging technology, with great potential for the development of clinical applications in diagnosis and surgical guidance.

## 4. Emerging Opportunities

### 4.1. Bacteria Identification

Bacterial identification in otitis media can provide important clinical information on whether antibiotics are needed and which type of antibiotics to initiate, depending on the type of bacteria detected. Raman spectroscopy and Raman imaging systems have been shown to have the potential to identify different bacterial strains within in vitro settings [42]. A proof-of-concept study utilized Raman microspectroscopy with a sparse multinomial logistic regression machine learning algorithm to study the major Raman markers for the three major bacteria species responsible for acute otitis media, *Haemophilus influenzae*, *Moraxella catarrhalis*, and *Streptococcus pneumoniae*. The study was able to validate their classification model using middle-ear effusion samples from patients with a 97% accuracy [43]. One feasibility study investigated a combined approach of low-coherence interferometry (LCI) and Raman microspectroscopy (RS) in a point-of-care setting for the identification of bacterial pathogens. Multiple validation experiments using cultured bacteria strain samples mimicking an in vivo environment were performed using this system and showed promising results when identifying and differentiating between *Pseudomonas aeruginosa* and *Streptococcus pneumoniae* [44]. Additionally, a multimodal handheld probe based on Raman spectroscopy and OCT has been reported [45]. Locke et al. demonstrated the feasibility of this bimodal imaging system to identify and differentiate between the four main pathogens for otitis media (*Haemophilus influenzae*, *Streptococcus pneumoniae*, *Moraxella catarrhalis*, and *Pseudomonas aeruginosa*) based on the refractive index, optical properties, and biochemical composition. All these imaging systems have huge potential to provide objective data to guide antibiotic selection in otitis media.

### 4.2. Artificial Intelligence and Machine Learning Opportunities

Machine learning (ML) is an AI application that can apply algorithms to make predictions on new data based on observations and analyses from previous datasets. One of the most common applications for ML and AI is a classification algorithm [46]. In recent years, AI has been shown to greatly enhance our ability to diagnosis middle-ear conditions such as preoperative cholesteatoma based on traditional imaging methods such as CT and MRI [47]. More importantly, many studies also have reported automated machine learning approaches to classify and triage images from traditional white-light otoscopy in the field of otolaryngology to improve diagnostic success rates [48,49,50,51,52]. The advancement in AI and ML algorithm research is particularly exciting, given that there is also great potential for the use of similar algorithms and models to improve the accuracy of these diagnostic classifications in middle-ear diseases to better guide treatment using the spectral and imaging information collected by label-free optical systems.

A few studies have developed ML/AI algorithms for label-free imaging. One example is the development of a machine learning approach in combination with SWIR for the diagnosis of otitis media with effusion. Kashani et al. presented the process of developing an ML algorithm from a total 1179 frames of SWIR otoscopic images sampled from 30 patients with a final accuracy of 90% to identify middle-ear effusions [24]. Monroy et al. reported an automated classification platform in combination with OCT imaging that was designed to identify biofilms and middle-ear effusion, providing a probable diagnosis. This ML algorithm was found to have an accuracy of over 90% [53]. The same group recently supplemented their automated ML algorithm with OCT images from a gold-standard animal model in addition to their original human pediatric patient image database, which increased the accuracy to 95% [54]. Cavalcanti et al. enhanced a smartphone-based multimodal imaging system with an ML algorithm that was able to identify otitis media with an accuracy of 79% [19]. Future endeavors in establishing larger databases of images collected via label-free optical technology in larger and more diverse patient populations can help to increase the power and predictive ability of such ML algorithms.

## 5. Clinical Translational Considerations

### 5.1. Integration with Clinical Devices: Practical Considerations

When adapting label-free imaging for clinical devices targeting middle-ear disease, there are a few practical considerations. For outpatient office visits, a handheld portable device that can provide real-time feedback about the imaging results is crucial. The long, narrow, and tortuous ear canals present challenges when designing a probe that is able to provide an adequate laser beam to reach the tympanic membranes of various patient populations without causing too much discomfort for patients. A device with a variety of probe-tip sizes and adjustable working distances may be solutions to accommodate such differences. A handle with a head able to be tilted at various angles may also be an explorable option [26,29]. For intraoperative guidance, such as for cholesteatoma surgery, contamination of the field view with blood and/or mucous tissue is an important interference to take into consideration when designing systems to reduce the signal-to-noise ratio.

### 5.2. Quality Control of Spectroscopy and Imaging Data

Consistent and reliable data are key for clinical translations. For many of the handheld devices introduced above, the physician or technician is responsible for holding the device steady while locating the tympanic membrane. Therefore, better algorithms are required to reduce the signal-to-noise ratio to achieve stability of the imaging data [25]. Better camera systems will also be able to capture other signals from areas with brighter chromophores and areas with weaker fluorescent biomarkers [40]. The variable results from different setups are also a major barrier to obtaining consistent data across the field. Greater consistency and standardization between different devices and system setups are also desired in order to achieve high-fidelity clinical data for applications.

### 5.3. Clinical Adoption

The current limitations for the diagnosis of middle-ear diseases using traditional otoscopes provide a great incentive for the clinical adoption of label-free imaging technology. Additionally, user-friendliness and convenience are crucial for wider adoption among physicians, providers, surgeons, technicians, and pathologists. Many preliminary studies have demonstrated prototypes that enhance traditional otoscopes with label-free imaging technologies such as SWIR and multicolor imaging [12,16,24]. This demonstrates the great compatibility of label-free imaging with existing devices routinely used in clinics and operating rooms. Researchers have also demonstrated a few portable handheld prototype devices for OCT [14]. The probe needs to be of a size small enough to not cause too much discomfort to patients. The ergonomics of these handheld portable devices should also be taken into consideration for future designs to facilitate a wide clinical adoption. Additionally, the imaging probes should be resistant to disinfection processes for daily usage with multiple patients.

The rapidly evolving ML and AI algorithms that allow for real-time automated classification will also greatly help with clinical adoption and application to increase user-friendliness for providers who are not imaging technology experts. The clinical community has shown a great deal of interest in adopting label-free technology for middle-ear diseases, as evidenced by the significant number of in vivo studies using label-free technology that have taken place in collaboration with clinicians and surgeons. Several startups based on label-free technology for middle-ear diseases have also emerged in recent years; clinical trials investigating these imaging devices are listed on ClinicalTrials.gov accessed on 16 January 2024.

Portability, ergonomics, ease of use, the ability to be sanitized, the automation of results, and compatibility with current medical devices are all key features for future developments of label-free technology for middle-ear diseases. Relevant evidence-based clinical guidelines on how to interpret data from these label-free imaging devices are also necessary to guide physicians who are not imaging experts in terms of diagnosis and treatment plans for middle-ear diseases.

### 5.4. Regulatory Challenges

New technologies require the regulatory approval of medical devices before application in clinical settings. Medical device approval from regulatory bodies such as the United States Food and Drug Administration (FDA) require demonstrations of safety, efficacy, and clinical benefits, typically in the forms of laboratory testing and clinical trials with human subjects. To date, Otosight is the first FDA-cleared OCT-based middle-ear imaging technique, which has been developed by PhotoniCare. Additionally, several clinical trials based on label-free techniques such as OCT and spectroscopy are currently active and listed on clinicaltrials.gov.

### 5.5. Democratization

To maximize the full potential of label-free imaging techniques, the cost must be reasonable to allow for adoption in various clinical setting across the world, not limited to a few hospitals and clinics in developed countries. We believe developing cost-effective label-free imaging devices for middle-ear diseases can democratize their use. This is especially beneficial in areas of the world where access to an otolaryngologist/ENT (ear, nose, and throat) specialist is very limited. Such device could provide valuable clinical information to aid general practitioners and healthcare providers to evaluate and treat middle-ear diseases. Ultimately, greater technical innovation and widespread clinical adoption will drive a reduction in cost and further promote wider use.

## 6. Conclusions

Over the past few decades, some efforts have been made to push label-free optical technology as a clinical tool across the broad spectrum of diagnostic, surgical, and pathological applications in the middle ear. Technological developments and the availability of detector technology in SWIR, OCT, DRS, Raman spectroscopy, and AF imaging coupled with advanced algorithms for data analysis would enable pushing this option forward to address unmet medical problems ranging from middle-ear fluid classification and quantifying to blood analyte monitoring in the tympanic membrane and in intraoperative settings. Despite this progress, the key challenges outlined in the above section must be tackled through close collaborations with otolaryngologists, biomedical engineers, and spectroscopists for clinical utility. The emergence of advanced AI algorithms is expected to accelerate the use of label-free optical technologies to address some of the most complex clinical questions. Further technological advances that can yield a high signal-to-noise ratio with minimal light-excitation power in less time are worthy of investigation to realize the dream of optical technologies being used in clinical settings. While the challenge remains substantial in translating the technology to clinics, recent advancements provide valuable insights and encouragement to resolve some of the most challenging hurdles, thereby paving the way forward for this ambitious goal.

In conclusion, this perspective article has reviewed the clinical applications of emerging label-free technologies over the past decades. The technology has already provided ample proof to demonstrate its clinical potential, with significant scope for further advancements. We believe the technological innovation has shortened the “translational gap” between knowledge gained from biomedical research and device development. We envision that these optical technologies would have great utility in investigating disease and abnormalities in one of the most inaccessible and fragile organs, the middle ear.

## Figures and Tables

**Table 1:** Summary of current applications of label-free imaging technology in middle-ear disease in ex vivo and in vivo settings.

Application Setting	Application Area	Technology	Reference
Ex vivo	Middle-ear tissue diagnosis	Raman spectroscopy	[7,8]
		Autofluorescence imaging	[9,10,11]
	Middle-ear fluid detection and differentiation	SWIR imaging	[12]
		Raman spectroscopy	[13]
	Tympanic membrane visualization	OCT	[14,15]
		SWIR imaging	[12]
In vivo	Tissue differentiation and visualization of tympanic membrane	Multicolor or multispectral autofluorescence imaging	[16,17,18,19]
		SWIR imaging	[12]
		OCT	[20,21]
	Otitis media	DRS	[22]
		SWIR	[23,24]
		OCT	[25,26,27,28,29]
		Multimodal	[30]

## Data Availability

No new data were created or analyzed in this study. Data sharing is not applicable to this article.
